# Periodical capacity setting methods for make-to-order multi-machine production systems

**DOI:** 10.1080/00207543.2014.886822

**Published:** 2014-02-24

**Authors:** Klaus Altendorfer, Alexander Hübl, Herbert Jodlbauer

**Affiliations:** ^a^ Department of Operations Management, Upper Austria University of Applied Sciences, Steyr, Austria.

**Keywords:** periodical capacity setting methods, demanded capacity, provided capacity, customer required lead time, service level, tardiness

## Abstract

The paper presents different periodical capacity setting methods for make-to-order, multi-machine production systems with stochastic customer required lead times and stochastic processing times to improve service level and tardiness. These methods are developed as decision support when capacity flexibility exists, such as, a certain range of possible working hours a week for example. The methods differ in the amount of information used whereby all are based on the cumulated capacity demand at each machine. In a simulation study the methods’ impact on service level and tardiness is compared to a constant provided capacity for a single and a multi-machine setting. It is shown that the tested capacity setting methods can lead to an increase in service level and a decrease in average tardiness in comparison to a constant provided capacity. The methods using information on processing time and customer required lead time distribution perform best. The results found in this paper can help practitioners to make efficient use of their flexible capacity.

## 1 Introduction

High service level and low tardiness are two important objectives of production planning and control in make-to-order (MTO) manufacturing systems. In a stochastic environment, practitioners deal with process- and customer uncertainties to set the capacity and to fulfill these objectives.

For the design of capacity setting methods, one key aspect is the available information about the stochastic environment of a production system. Even if information about stochastic customer required lead time and stochastic processing times is available, practitioners can still decide how much information to use for the capacity setting decision. Such information can be delivered by enterprise resource planning systems, production data acquisition systems and/or manufacturing execution systems. However, information processing to ensure its applicability for decision models often leads to additional efforts or costs.

When a company can forecast the demanded capacity, the provided capacity of the production system has to be set based on the demanded capacity of the customer. Such capacity adjustment is enabled by flexible working contracts and capacity accounts which become more and more applied in manufacturing companies. Upper and lower bounds for the provided capacity are introduced because capacity is for companies not arbitrary changeable due to e.g. law for working times, contract workers and investment/de-investment in machinery and equipment. These tools allow companies to deal with customer demand uncertainty by adjusting their capacities. Furthermore, the capacity cannot be adjusted instantaneously but a periodic capacity adjustment is allowed. This reflects the situation of a rolling horizon planning in real production systems where capacity can, for example, be defined for the next week on a daily basis taking into account the currently known orders.

Based on the assumption that processing time distribution and customer required lead time distribution are known, these two information sources are used to develop different capacity setting methods which are tested in a discrete event simulation study. Since many manufacturing companies are dealing with bottlenecks in the production process, a single-machine framework is considered in this paper, which is finally extended to a multi-machine setting.

In this paper a periodical decision support for short and medium term capacity setting to improve service level and tardiness is developed. Stochastic customer behaviour (information uncertainty: not all information is available when the decision is taken), the integration of stochastic processing process (process uncertainty: the processing time of orders is not deterministic), and the rolling horizon effects of a planning system (re-evaluation effects: decisions taken in the past influence the current decision space and not the full length of the decided plan is really implemented) are considered by the different methods.

The paper is organized as follows. In Section 2 the relevant literature and the contribution of the current paper is presented. Section 3 outlines the model framework based on the single-machine setting. The model for periodical capacity setting methods are presented in Section 4. In Section 5 the model is extended to a multi-machine production system. A simulation study for illustrating the developed methods is given in Section 6, whereby the results are presented in Section 7. The paper concludes with a short summary and further research.

## 2 Literature review

On the one hand the due dates of the customer can be negotiated to create a more smooth capacity demand (Corti, Pozzetti, and Zorzini 2006; Hopp and Roof Sturgis 2000; Hegedus and Hopp 2001; Keskinocak and Tayur 2004). On the other hand the capacity can be adjusted according to the fluctuations of the customer demand (Bradley and Glynn 2002; Buyukkaramikli, Bertrand, and van Ooijen 2013; Kok 2000; Li, Hendry, and Teunter 2009; Mincsovics and Dellaert 2009; Van Mieghem and Rudi 2002). The methods discussed in this paper are based on capacity adjustment literature whereby a flexible capacity with upper and lower bounds is assumed. Therefore, the capacity adjustment literature stream is focused in this literature review.

Capacity expansion problems have firstly been studied in capacity investment literature (Chenery 1996; Kok 2000; Luss 1982; Manne 1961; Pibernik and Yadav 2009). Chenery (1996) and Manne (1961) assume deterministic increasing demand and whenever demand reaches available capacity the capacity is expanded. Manne (1961) included probabilities instead of a constant rate of growth in demand and backlogs to the model of Chenery (1996). Luss (1982) conducted an extensive literature review about capacity expansion problems. They classified capacity investment problems in several categories emphasizing modelling approaches and algorithmic solutions. Segerstedt (1996) developed a capacity constrained multi-stage inventory and production control problem. Segerstedt (1996) minimizes the inventory costs and shortage cost, whereby the cumulated capacity concept is applied as constraint. The cumulated demanded capacity is not allowed to exceed the cumulated provided capacity. Kok (2000) compared two capacity allocation strategies. A fixed capacity is assumed and if the demand exceeds the fixed capacity the orders are delayed. Moreover, an additional capacity is introduced by hiring personnel.

Decision problems with capacity expansion and/or reduction are modelled in most cases as dynamic programs (Bradley and Glynn 2002; Li, Hendry, and Teunter 2009; Van Mieghem and Rudi 2002). Bradley and Glynn (2002) developed an analytic model for a single-machine and single product system which describes the optimal long term balance between capacity and inventory. They show that optimal inventory policy varies with capacity investment and that higher capacity invested allows less inventory. Moreover, the authors describe how inventory should be optimally substituted for capacity to minimize costs when the capacity level varies. Van Mieghem and Rudi (2002) addressed this issue for a more general situation and gained similar results. Obviously there is a trade off between capital invested in capacity and costs of the employed capital in inventories. In Li, Hendry, and Teunter (2009), capacity allocation methods with mixed integer programming methods are compared for supply chain optimization. The authors identified that an integrated planning approach achieves better results than an approach where each sub problem is treated separately.

The MTO ability of production systems is evaluated in Jodlbauer (2008) depending on the provided capacity, the customer required lead time distribution and the demand fluctuation. The result of this evaluation shows that applying a capacity adjustment method, which enables the reaction on short term peaks, can decrease the demanded capacity for MTO environment. In Jodlbauer and Altendorfer (2010) a concept for optimizing the overall provided capacity is presented which again uses the customer required lead time distribution. The result of this paper indicates that flexible capacity on a short and medium term basis can lead to a cost decrease.

The papers of Balakrishnan, Patterson, and Sridharan (1996) and Balakrishnan, Patterson, and Sridharan (1999) discuss the capacity rationing problem for a two product production system whereby one product class leads to higher profits per unit. A order rejection policy for the lower profit products to maximize company profit is illustrated. In Kok (2000), capacity allocation is discussed where capacity has to be allocated to different product groups while minimizing a total cost function. In this model, the production is triggered by an order-up-to policy for each product group.

In recent research queuing state dependent capacity adjustment models focusing on the transient behaviour of the queuing system when switching between different capacity levels are studied (Buyukkaramikli, Bertrand, and van Ooijen 2013; Mincsovics and Dellaert 2009). In Mincsovics and Dellaert (2009) a continuous setting is discussed in which an up-switching-point and a down-switching-point are identified and each switch incurs costs. A periodic setting with two possible capacity levels has been studied extensively in Buyukkaramikli, Bertrand, and van Ooijen (2013).

In this paper a cumulated capacity concept is applied, which is used by Segerstedt (1996). The M/M/1 queueing state dependent capacity adjustment models of Mincsovics and Dellaert (2009); Buyukkaramikli.2011 are extended by general distributions for the arrival process and processing times. Moreover, the capacity setting method is extended to a multi-machine production system. Our framework also includes a distribution of the customer required lead time. Therefore, Jodlbauer (2008) is extended by an rolling horizon and is implemented for calculating the provided capacity. A limitation of the presented approaches is that no analytical evaluation of their performance is possible and therefore simulation is applied. After determining the provided capacity, in a next step, a scheduling approach could be considered which is left for further research.

## 3 Model framework

**Figure 1.**
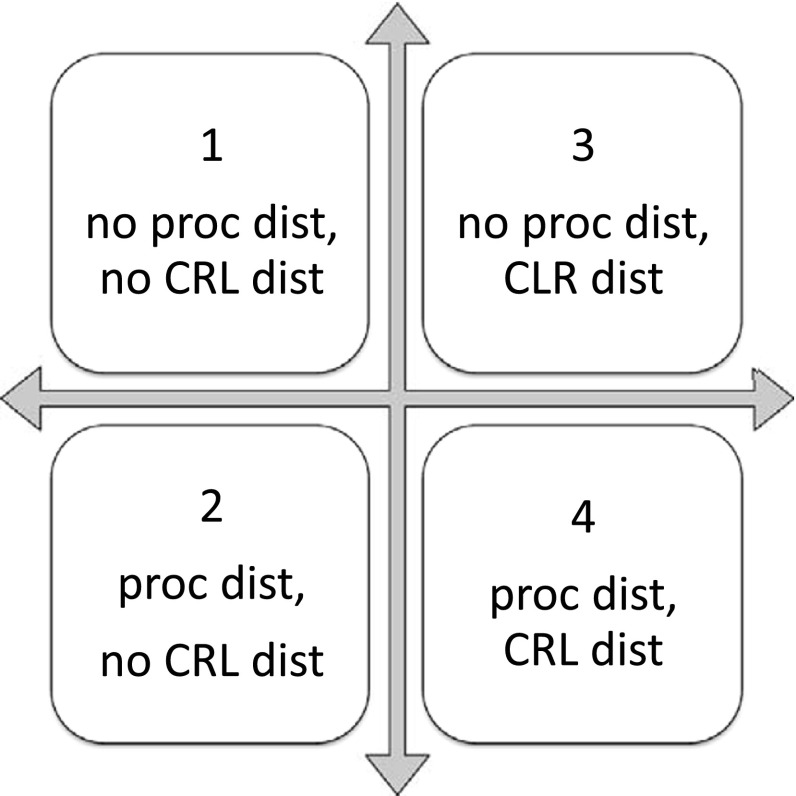
Matrix of stochastic information for demanded capacity – four methods.

Since information processing may lead to additional effort and costs, a set of methods with different information levels is developed for approximating the demanded capacity as illustrated in Figure 1. The matrix indicates the two dimensions processing time distribution (proc dist) and customer required lead time distribution (CRL dist). The decision maker can include or not include information of the process uncertainty or customer required lead time uncertainty by using the corresponding distribution. Quadrant a and b apply no additional information for processing time uncertainties whereby quadrants c and d include the processing time distribution. Customer required lead time uncertainties are implemented in methods b and d whereby quadrants a and c are using no information about the customer required lead time distribution.

**Figure 2.**
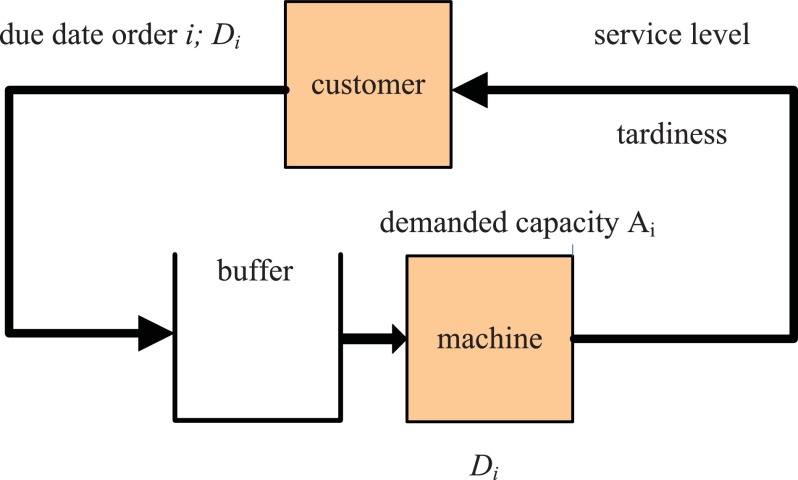
Single-machine production system.

Figure 2 shows the basic model where order 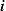 with the due date 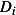 is stated by a customer. The customer required lead time represents the time gap between the point of time where the order is stated and the due date of the customer order. The production system can either fulfil the customer order on time or the customer order is delayed. If an order is satisfied earlier then it is considered to be on time. This circumstance is measured by the two metrics service level and tardiness. No lost sales are considered in the model. If the machine is idle the order is released into production, otherwise the order is waiting in the buffer for processing. Only one order can be processed at a time. Customer required lead times and processing times are assumed to be stochastic.

The production system is assumed to work on a MTO basis. This means production orders are created based on already known demand and not based on forecast. Only the capacity demand is forecast in some of the following methods using the distribution of customer required lead time.

## 4 Model

In Table 1 the variables and in Table 2 the parameters for the single-machine production system are presented.

**Table 1.** Definition of variables for single-machine model.

Notation	Description
	Random variable for due date of order *i*
	Random variable for pushed up due date of order *i*
	Random variable for demanded capacity of order *i*; is the current backlog
	Random variable for cumulated demanded capacity until time *t*
	Random variable for pushed up cumulated demanded capacity until time *t*
	Random variable for capacity weighted customer required lead time of all orders
	Expected value and variance of a random variable
	CDF (cumulated distribution function) of random variable *G*
	Inverse of the CDF of random variable *G*
	Variable for approximating cumulated demanded capacity at the machine at time *t* based on method
	Variable of bounded provided capacity after each capacity setting cycle based on method
	Variable of unbounded provided capacity after each capacity setting cycle based on method
	Variable for capacity provided in past period
	Variable for capacity account

 

**Table 2.** Definition of parameters for single-machine model.

Notation	Description
	Method parameter of cumulated demanded capacity:
	: Deterministic processing times; no CRL distribution
	: Processing time distribution; no CRL distribution
	: Deterministic processing times; CRL distribution
	: Processing time and CRL distributions
	Method parameter of provided capacity:
	: Full utilization
	: Maximum safety
	: Service level target
	Parameters for lower/upper bound for provided capacity
	Parameter for period of time for which is set
	Parameter for time where the system has (re)started
	Parameter for average provided capacity per period
	Service level parameter for capacity setting method
	Capacity feasibility parameter for capacity setting methods and
	Operations characteristic of the machine
	Parameter for evaluation time window

**Figure 3.**
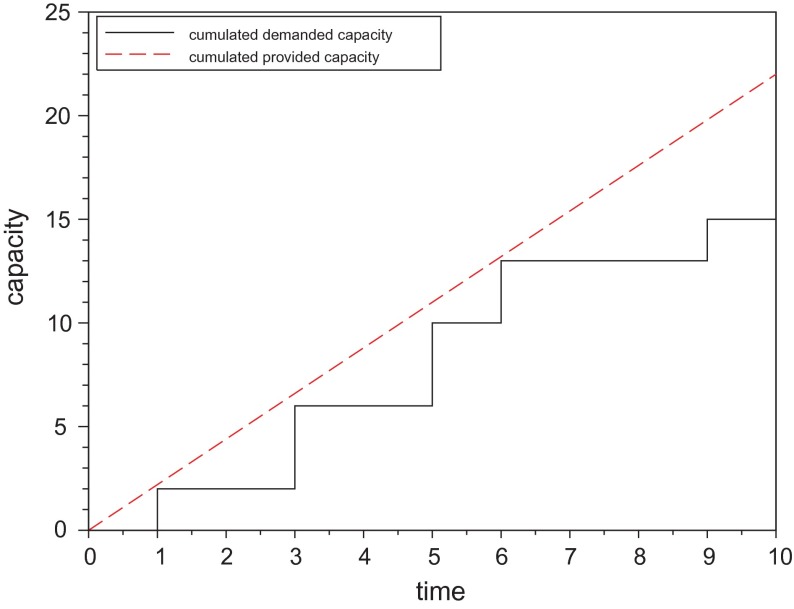
Basic idea of capacity setting.

To produce all orders on time, the demanded cumulated capacity 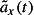 has to be lower than or equal to the cumulated provided capacity 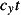 at all points in time as illustrated in Figure 3. A combination of 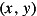 (see Table 2) is defined as periodical capacity setting method, whereby 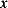 and 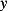 indicate the used methods for demanded and provided capacity respectively.

**Figure 4.**
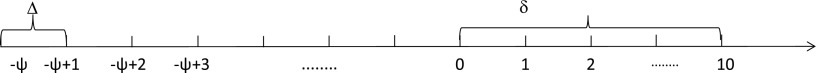
Timeline.

The capacity setting is performed each 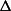 periods for an evaluation time window of 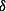 periods in the future as shown in Figure 4. Compared to scheduling approaches or medium term production planning like MRP, 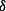 corresponds to the planning horizon.Hence, the capacity setting is based on the already stated customer orders. For 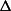 being one week and 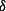 being two weeks, for example, the capacity setting would be done weekly taking into account the known orders for the next 10 working days. Without loss of generality, the current time of each periodical capacity setting cycle is set to zero.

The periodical capacity setting methods follow three steps:

(1) Approximate the cumulated demanded capacity 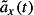 required by the customers; four methods are presented.
(2) Calculate unbounded provided capacity based on the demanded capacity; 3 methods are presented.
(3) Set capacity based on the capacity account.

### 4.1 Approximation of demanded capacity (Step 1)

**Figure 5.**
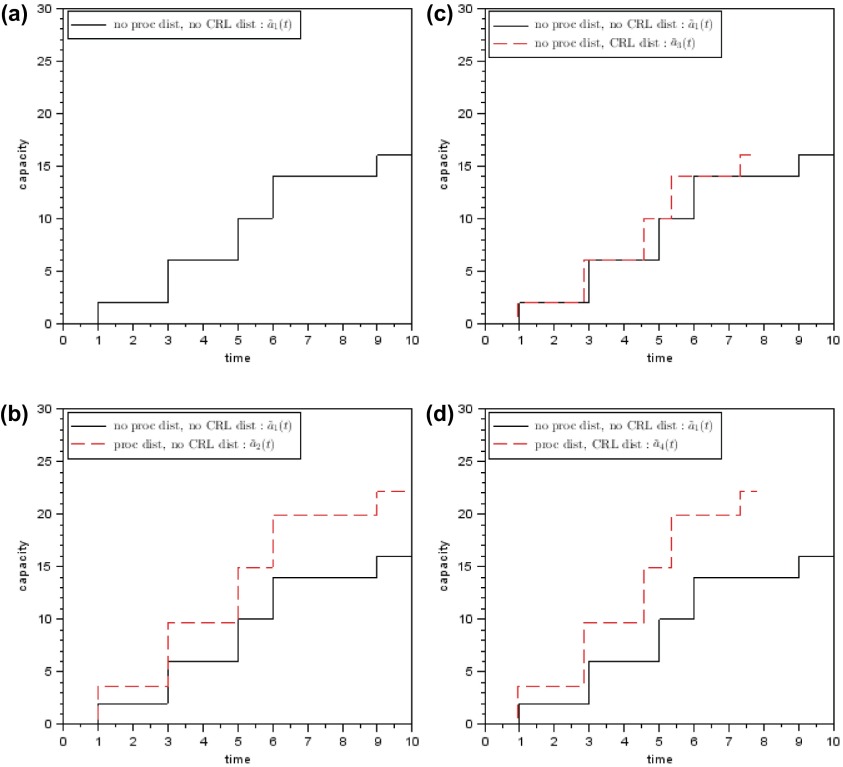
Cumulated demanded capacity – four methods.

In the following section the first step of the periodical capacity setting methods is detailed. Four methods for approximating the cumulated demanded capacity are formally defined and illustrated in Figure 5.

#### Deterministic processing times, no CRL distribution: 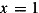


4.1.1 

Each order 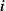 requires a capacity 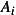 at due date 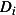. In this case 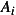 is approximated with its expected value 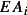 without any additional information. This means the decision maker has no information about the process- and demand uncertainty or does not want to invest additional effort for information processing. Equation 1 cumulates the demanded capacity up to time 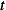 which is illustrated in Figure 5(a):1 
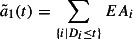



#### Processing time distribution, no CRL distribution: 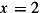


4.1.2 

Method 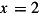 includes the information of the processing time distribution but uses no information about the customer required lead time distribution to approximate a cumulated capacity demand. A higher amount of demanded capacity results from this approximation in comparison to method 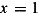 as indicated in Figure 5(b) (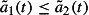) due to a safety capacity for the processing time distribution.

The random variable of the cumulated demanded capacity is defined in Equation (2):2 
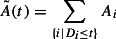
Note that Equation (2) includes 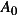 since 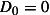. In Equation (3), the demanded capacity to fulfil a set of orders (until time 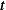) with probability 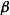 (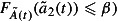 is calculated.3 
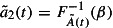
Processing times may not be deterministic due to products requiring different amount of machine capacity, stochastic processing speed, set up time or down times. In this case, the realization of the demanded capacity will exceed the expected value 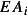 with a certain probability. If, for the example, 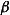 is set to 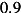, then method 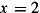 includes safety capacity to fulfil the stochastic capacity demand with 90% probability. This method works for all distributions of the demanded capacity where a quantile function exits.

The safety capacity of method 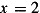 in comparison to method 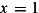 is 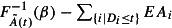, which is the difference between the dashed and solid line in Figure 5(b).

#### Deterministic processing times, CRL distribution: 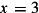


4.1.3 

The customer required lead time distribution is used for evaluating method 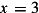, which results in an earlier allocation of the demanded capacity compared to method 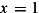 to forecast short term orders which are not yet known. Method 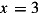 still assumes deterministic processing times as 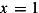.

**Figure 6.**
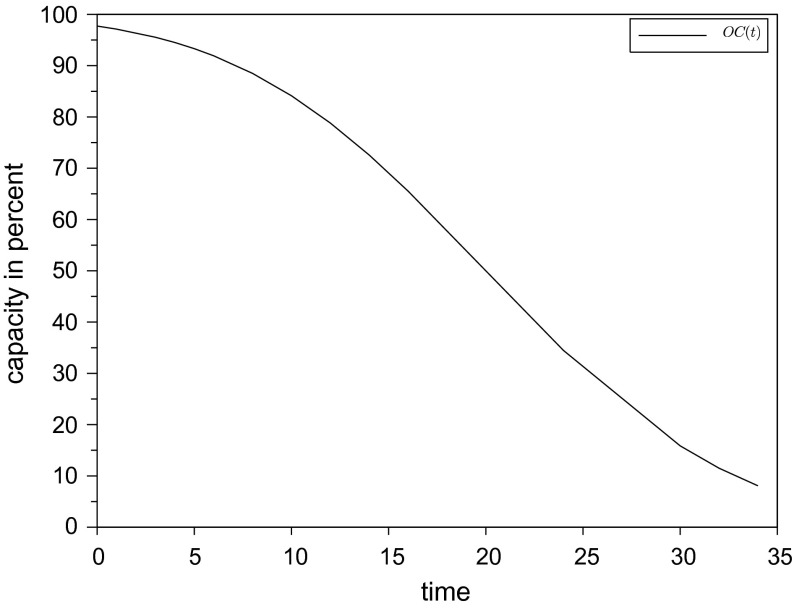
Operations characteristics.

The customer required lead time is defined as the time between the due date of an order 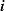 and the point in time when this order is stated. At a certain point in time, the demanded capacity is defined by the already stated customer orders. However, according to the customer required lead time distribution, additional demand – defined as anticipated demand – can be requested by customers. To include the anticipated demand, the concept of the operations characteristic (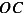) is applied, whereby this 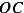 defines the relationship between the customer required lead time and the demanded capacity at a machine (Jodlbauer 2008). For a single-stage model, the 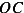 shows how much of the customer required capacity is known how many periods in advance. Figure 6 shows an example of an 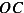 where 50% of demanded capacity is known 20 periods in advance.

In Jodlbauer (2008), the 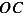 concept encompasses the concept of a constant remaining processing time. This remaining processing time can be the transportation time to the customer in a single-stage production system or the remaining processing and handling time in a multi-stage production system. Since this time is assumed to be constant in Jodlbauer (2008), which does especially for job shop production systems not hold, the 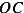 concept is slightly adapted in this paper.

The customer required lead time is weighted by its capacity consumption so that the capacity weighted customer required lead time 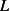 results (Jodlbauer 2008; Jodlbauer and Altendorfer 2010). Finally, the OC is calculated in Equation (4):4 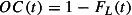
Based on the OC, it is necessary to produce the already stated orders earlier to have enough safety capacity left for the anticipated future demand. Therefore, the pushed up due date 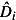 is used instead of the customer required due date 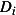. The following Equation (5) for approximating the cumulated demanded capacity can be stated:5 
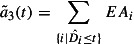
Equation (6) shows how the due date of each order based on the 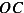 is pushed up to ensure some safety capacity for the anticipated demand (therefore 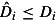 holds).6 
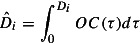
In this method not a typical forecasting approach is conducted where the customer demand of a certain product or a certain product group is forecast, but only the capacity demand still to occur on the short and medium time range is anticipated for a certain machine based on past data.

#### Processing time and CRL distributions: 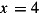


4.1.4 

In method 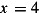, the concepts of methods 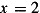 and 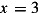 are combined. Therefore, method 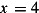 uses the information of the customer required lead time distribution and the processing time distribution. Again, a certain probability 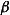 is used to calculate the cumulated demanded capacity based on the processing time distribution.7 
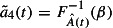
However, the random variable of pushed up cumulated demanded capacity until time 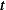 is defined in Equation (8), because method 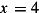 also includes the anticipated demand, where the due date of each order is pushed up according to Equation (6).8 
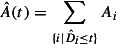
Method 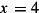 pushes up the demanded capacity (based on the customer required lead time distribution) and increases it according to the processing time distribution in comparison to method 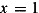 (see Figure 5(d)).

### 4.2 Setting the unbounded provided capacity (Step 2)

**Figure 7.**
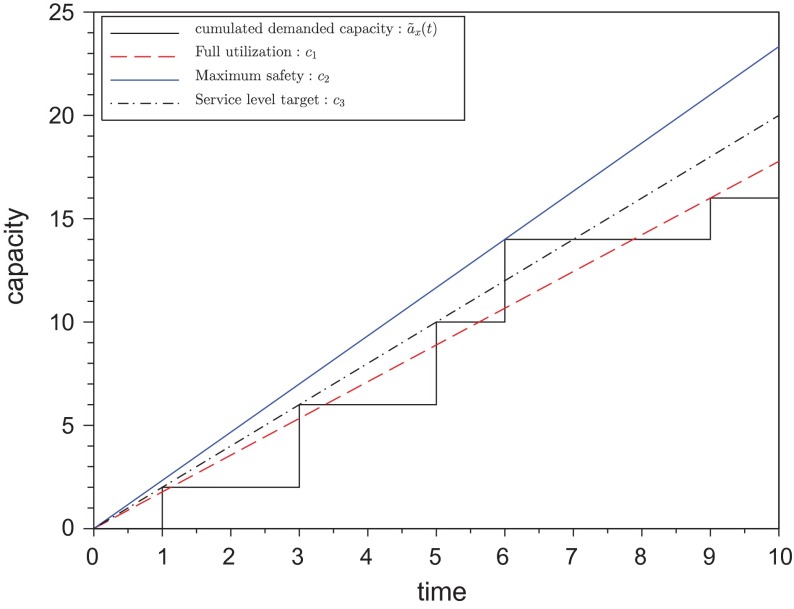
Provided capacity.

Based on the cumulated demanded capacity, calculated with one of the methods 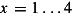, the unbounded provided capacity is set. Three methods for calculating the provided capacity are presented in this section and illustrated in Figure 7. 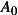 – the current capacity backlog of the system – is in Figure 7 set to zero.

The provided capacity for method 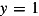 is set to the cumulated demanded capacity at the end of the evaluation time window 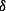. Method 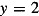 is based on the maximum increase of the cumulated demanded capacity 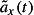. Finally, method 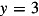 uses a kind of service level 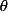 to relax the constraint of method 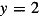, so that not each order has to be ready on time.

#### Full utilization: 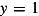


4.2.1 

For this capacity setting method the provided capacity is set to the average demanded capacity within an evaluation time window 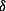. Therefore, the cumulated demanded capacity at the end of the evaluation time window is divided by the time where the last customer order reaches its due date. Assuming the approximated demanded capacity occurs, this method ensures full utilization for the machine, because the provided capacity is set to the cumulated demanded capacity for observed evaluation time window 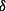. Note that methods 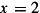 to 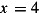 lead to a safety capacity which will often not be utilized and therefore the method 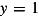 will in these cases not lead to 100% utilization.9 
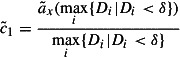
The maximum over all 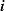 is responsible for searching the due date of the last order within the evaluation time window

#### Maximum safety: 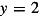


4.2.2 

Method 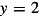 works as follows: a straight line starting at zero with minimum slope being greater or equal than the cumulated demanded capacity is constructed. The fraction in Equation (10) can be interpreted as the slope of cumulated capacity demand at due date 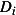. The 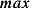 operator identifies the maximum slope over all due dates 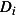 leading to a maximum safety in capacity provided. As an assumption at least one order has to be in the system. Note that capacity backlogs are ignored since they would lead to an infinite increase, however, they are included in 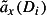 when the first due date is evaluated.10 
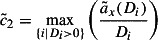



#### Service level target: 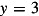


4.2.3 

Since it could be too ambitious to set the provided capacity to the maximum increase as calculated in Equations (10) and (11) presents a smoothed version of this approach, where not the whole capacity demand has to be finished on time. The lowest 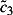 which still satisfies a service level 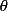 for the evaluation time window 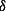 is searched in the following optimization problem:11 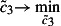


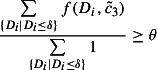
The function 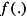 defined in Equation (12) delivers one if a due date (one or more orders can have the same due date) is on time and zero if a certain due date does not hold. 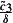 is the slope of the provided capacity.12 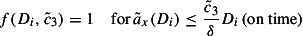


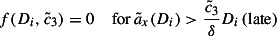
If 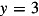 is applied in combination with methods 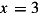 and 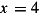, 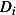 has to be substituted by 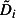 for Equations (11)–(12), because the customer required lead time distribution is included in the approximations.

### 4.3 Capacity account (Step 3)

The bounded provided capacity 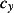 is introduced for the developed capacity setting methods. If the unbounded provided capacity 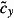 is below 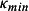 then the provided capacity is set to 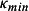 and if the candidate exceeds 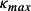 then the provided capacity is set to 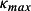. Equation (13) corresponds to the flexible capacity range that can be provided in the production system.13 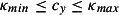
Since most flexible working time contracts have a defined average working time 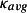, a capacity account 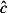 is introduced and Equation (14) is added to ensure that the provided capacity is on average lower or equal than the allowed average working time. When the capacity account 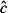 is positive (e.g. 16 h), the production system has some capacity which can be provided above the average working time (e.g. 2 shifts with 8 h each on Saturday). All periods since the system has (re)started are taken into consideration which leads to Equation (14) with 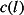 being the capacity provided in past period 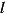. If the production system has collected a positive capacity account by setting the capacity less then 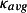 then the capacity can be set to a level exceeding 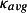 if necessary.14 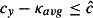


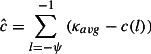
The candidate for provided capacity 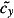 from methods 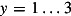 is constrained by Equations (13) and (14) in the following Equation (15).15 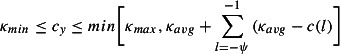
According to the procedure of periodical capacity setting methods, steps 1 to 3 are repeated each 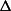 periods to implement the rolling horizon planning effect.

## 5 Multi-machine concept

In this section the basic single-machine production system is extended to a multi-machine production system. The methods can be applied for any arbitrary production system structure according to the respective routing file. Order 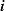 with the due date 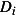 is stated by a customer. On each machine 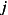 a demanded capacity 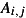 is requested by the order 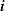.

To implement the methods developed for the single machine case also for a multi-machine setting, an order due date for each machine 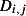 has to be identified. This order due date is based on the customer order due date applying a backward scheduling similar to the MRP (material requirements planning) algorithm (Hopp and Spearman 1996). The difference to MRP is that for the capacity setting methods no production plan is generated but only the capacity demand has to be identified and therefore no or only little safety time is included in this backward scheduling.

Based on these 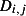 values, also the customer required lead times have to be adapted, and therefore in the multi-machine concept 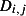, 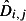, 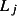 and 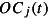 replace 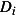, 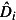, 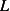 and 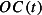 respectively in Equations (1)–(15) as indicated in Table 3. Moreover, the demanded capacity for a machine within the multi-machine production system needs machine index 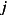 to be added and therefore 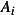 is replaced by 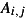. Also the lower and upper bound for the provided capacity 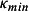 and 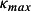 are defined for each machine 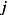.

**Table 3.** Definition of additional variables for multi-machine model.

Variable	Description
	Random variable for machine depended due date of order at machine .
	on the last machine is equal to stated by the customer
	Random variable for pushed up due date of order at machine
	Random variable of remaining capacity demand for order after machine
	Set of machines for remaining processing steps of order after machine
	Random Variable for demanded capacity of order at machine
	Random variable for capacity weighted customer required lead time at machine
	OC of machine

This backward scheduling is introduced in Equation (16):16 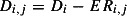
In Equation (17) the remaining capacity demand for order 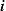 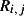 is defined, whereby only the demanded capacity at the remaining machines indicated by 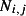 for finishing an order are considered. The parameter 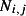 represents the set of machines for remaining processing steps of order 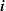 after machine 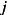 known from the respective routing file. Assuming for the multi-machine example presented in Section 6, that the current machine is machine three, 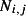 consists of machine four and five since these processing steps have to be finished after processing at machine three.17 
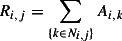
For methods 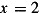 and 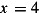 two alternatives 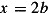 and 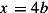 including the stochastic behaviour of remaining capacity demand are developed. Hence, the existing methods 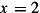 and 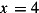 are defined in the multi-machine production system as 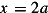 and 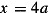.

In Equation (18) the CDF of 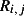 is used to calculate 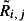 based on the distribution of the remaining capacity demand. Thus, 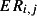 is replaced in Equation (17) by 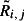.18 
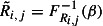
Comparing the sum over the means of the provided capacity in Equation (17) with the inverse of the CDF of the processing time distribution for the remaining processing steps of an order in Equation (18) leads to a difference which is equivalent to the safety time concept also applied in MRP. This safety time accounts for the possibility that a certain order has a higher processing time than planned at a certain machine. However, the approach presented in Equation (18) does not consider the queue length as discussed for example in the Workload Control (Bechte 1988) literature where also the queuing between the processing steps is considered. Compared to the safety time concept in MRP, which also includes some safety time for queuing, this safety time only includes the processing instabilities. This is assumed since only the workloads and their latest possible date 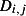 for each single machine are needed but no production plan with start dates is generated.


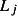
 is calculated in Equation (19) based on 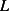. For simplification reasons in the calculation of 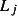, all 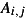 are assumed to have a common stochastic distribution and therefore 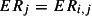 holds.19 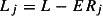
Note that this assumption only influences the 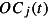 applied in the methods 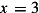, 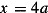 and 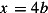.

## 6 Simulation study

The simulation study is conducted for comparing the behaviour of the presented methods to analyse which information improves the capacity setting methods. In this simulation study 19,500 simulation runs for the single machine production system and 28,500 simulations runs for multi-machine production system have been conducted as shown in Table 4.

**Figure 8.**
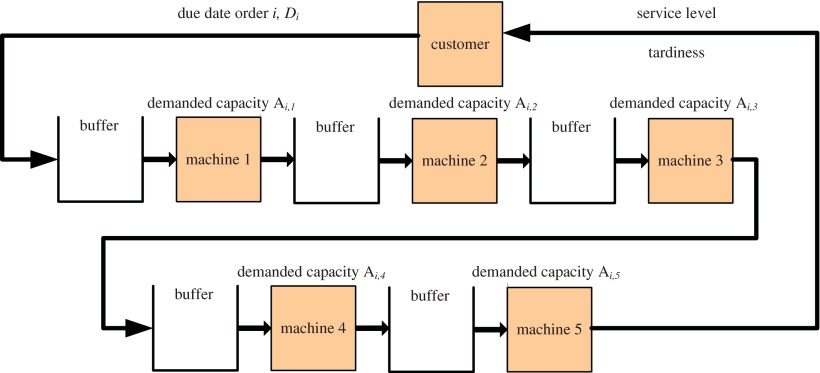
Multi-machine production system.

Five sequential machines are investigated for the multi-machine production system as illustrated in Figure (8). The used simulation framework is explained in more detail in Hübl et al. (2011). The simulation model has been validated as proposed in Kleijnen (1995) by checking the model behaviour of the single-machine case with constant capacity compared to developed analytic results.

**Table 4.** Simulation runs.

Description	Multi-machine	Single-machine
Planned utilization values	15	15
Scenarios	10	10
Methods for demanded capacity	6^a^	4^b^
Methods for provided capacity	3^c^	3^c^
Replications	10	10
Constant capacity	1	1
Simulation runs	28,500	19,500

a 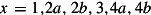.

b 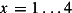.

c 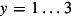.

The planned utilization is set to the same level for all machines. The following values for planned utilization are tested: 0.7, 0.75, 0.8, 0.85, 0.875, 0.9, 0.91, 0.92, 0.93, 0.94, 0.95, 0.96, 0.97, 0.98, 0.99.

Ten different scenarios for single and multi-machine setting are tested where parameters for processing time, customer required lead time, upper and lower bound for provided capacity and period for setting the provided capacity are predefined as shown in Table 5. A basic scenario is defined as the basis for all other scenarios with a variation coefficient for the processing time distribution of 1, mean customer required lead time of 20 time periods for the single-machine production system and 100 time units for the multi-machine production system. The upper and lower bound for the provided capacity is given by 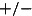 20% and 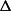 is set to 10 periods.

**Table 5.** Scenarios.

Description				
Basic	1	20/100	0.8, 1.2	10
High proc-time variation	1.5	20/100	0.8, 1.2	10
Low proc-time variation	0.5	20/100	0.8, 1.2	10
High cust. req. leadtime	1	50/250	0.8, 1.2	10
Low customer req. leadtime	1	10/50	0.8, 1.2	10
High flexibility	1	20/100	0.5, 1.5	10
Low flexibility	1	20/100	0.95, 1.05	10
Indefinite flexibility	1	20/100	0,	10
Long capacity setting period	1	20/100	0.8, 1.2	50
Short capacity setting period	1	20/100	0.8, 1.2	5

The periodical capacity setting methods are compared to a scenario where the production system provides constant capacity. The average of the provided capacity is for all tested scenarios equal, also for the constant provided capacity scenario. In the single-machine production system, the four methods 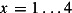 for identifying the demanded capacity are compared. For the multi-machine production system, the additional two methods for calculating the demanded capacity due to the machine depended due date are included (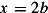 and 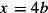). Therefore, six methods are compared in the multi-machine production system as shown in Table 4. Ten replications are produced for each parameter combination.

The following values are set: 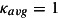 , 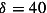 for single-machine production system, 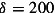 for multi-machine production system, 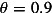 and 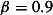. The parameters 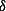, 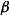 and 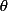 have been identified in preliminary studies to perform well with respect to service level and tardiness for the basic scenario at 80% utilization. The simulation runs for 500,000 time periods, a warm up period of 100,000 time units is excluded from the measured results and the system is restarted every 100,000 time units for data generation reasons.

Processing times are log-normal distributed and the distribution of the customer required lead time is implemented as exponential distribution for the simulation experiment. The distributions are chosen for non negativity. For simplicity reasons, the distribution of 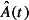 is assumed to be log-normal as well. The mean customer required lead time is differently assigned for the single-machine and the sequential production system. In the third column of Table 5, the first number is dedicated to the single-machine system and the second number to the sequential production system. The mean processing time per order is one period.

The interarrival time of the customer orders, which is used to influence the planned utilization, is also exponentially distributed. No set-up times and no transportation times are included in the simulation. The capacity setting methods for the provided capacity are implemented in the simulation model by changing the machine speed. Whenever the average machine speed (which is equivalent to the capacity account) is below or equal to one, the provided capacity can be set – if necessary – higher than one. The average machine speed is cleared at each system restart. Relating the simulation periods to one shift of a working day and assuming that two shifts per day is the working time for five days a week, then the capacity is set every week ( 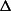 = 10 shifts). The capacity account in the beginning is zero. The simulation is conducted in AnyLogic 6.4.1.

## 7 Results

In the first step, the best capacity setting method 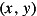 for the basic scenario is evaluated. Based on this evaluation, the results of the best method 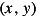 are compared to the situation with constant provided capacity for the two production systems. This is followed by an analysis over all defined scenarios. For all investigations, the performance measures service level increase and tardiness decrease in comparison to average capacity provided are used.

### 7.1 Evaluation of best method combination

Table 6 shows the best method combination 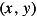 compared to constant provided capacity for 15 different planned utilization values. Tardiness and service level are treated separately for identifying the best method combination.

For the single-machine case the results for the planned utilization of 94% indicates an increase of 23% in the service level by the use of method 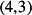. At the same planned utilization value, a decrease of 91% in average tardiness by the use of method 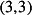 occurs. The results for the single-machine production system show that for most of the planned utilization values tested, the method 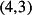 leads to the best results. For the multi-machine case the result from Table 6 shows a balance between methods 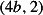 and 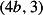.

**Table 6.** Service level increase and tardiness decrease potential in basic scenario.

Utilization	Single-machine case				Multi-machine case			
	*Service level*		*Tardiness*		*Service level*		*Tardiness*	
	Increase	Method	Decrease	Method	Increase	Method	Decrease	Method
[%]	[%]	(x, y)	[%]	(x, y)	[%]	(x, y)	[%]	(x, y)
70.0	1.0		18.0		0.0		9.0	
75.0	1.0		23.0		0.0		11.0	
80.0	1.0		28.0		1.0		13.0	
85.0	3.0		58.0		1.0		15.0	
87.5	6.0		72.0		2.0		16.0	
90.0	10.0		82.0		3.0		17.0	
91.0	12.0		85.0		3.0		17.0	
92.0	15.0		88.0		4.0		19.0	
93.0	18.0		90.0		4.0		20.0	
94.0	23.0		91.0		4.0		19.0	
95.0	27.0		94.0		5.0		19.0	
96.0	32.0		93.0		4.0		17.0	
97.0	37.0		95.0		5.0		20.0	
98.0	43.0		94.0		2.0		14.0	
99.0	44.0		85.0		0.0		16.0	

Notes: 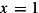: det proc times, no CRL dist; 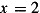: proc time dist, CRL dist; 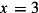: det proc times, CRL dist; 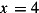: proc time dist, CRL dist; 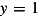: full utilization; 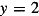: maximum safety; 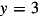: service level target.

**Table 7.** Comparison of methods over all scenarios.

Single-machine case			Multi-machine case		
Service level		Tardiness	Service level		Tardiness
Method	% of cases	% of cases	Method	% of cases	% of cases
Constant	9.3	8.0	Constant	14.0	9.3
	0.0	2.0		14.7	17.3
	16.7	14.7		8.7	4.7
	8.0	16.0		12.0	7.3
	66.0	59.3		14.0	15.3
	24.7	31.3		16.7	20.0
	14.0	14.0		20.0	26.0
	52.0	46.7		0.7	2.7
				50.7	58.0
				34.7	30.0

Notes: 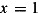: det proc times, no CRL dist; 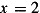: proc time dist, CRL dist; 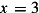: det proc times, CRL dist; 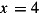: proc time dist, CRL dist; 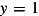: full utilization; 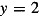: maximum safety; 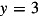: service level target.

As a measure for evaluating which method is the best for the two production systems, the percentage of simulation experiments dominated by a certain method is used, whereby only the average of a replication set is considered. Therefore, Table 7 summarizes the percentage of cases where the tested methods lead to the best results in service level increase and average tardiness decrease when all 10 scenarios are taken into account. In the multi-machine production system in 20% of all parameter combinations method 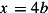 leads to the best result concerning service level increase.

The first result found in this study is that the same methods perform well concerning service level increase and average tardiness decrease. In the single-machine case the method 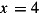 for demanded capacity, which uses both customer required lead time and processing time distribution, leads in about 60% of cases to better results for service level and tardiness. Only in about 8% of the cases does the constant provided capacity scenario lead to better results, which are discussed in the next subsection. The best method for provided capacity calculation is in the single-machine case 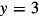, which leads in approximately 50% of cases to the best result over all scenarios.

For the multi-machine production system, the best result for calculating the demanded capacity is again found with both version of method 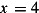, whereby a slight advantage for alternative 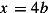 is identified. This means the best method again uses all the provided information about customer required lead time distribution and processing time distribution. Due to the small difference in the result between method 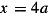 and 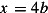 it is not possible to clearly specify whether it is better to have the machine dependent due date calculation based on expected values for processing times or based on the processing time distribution. In the multi-machine production system 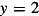 is the best method for defining the provided capacity.

### 7.2 Result comparison best method with constant provided capacity scenario

**Figure 9.**
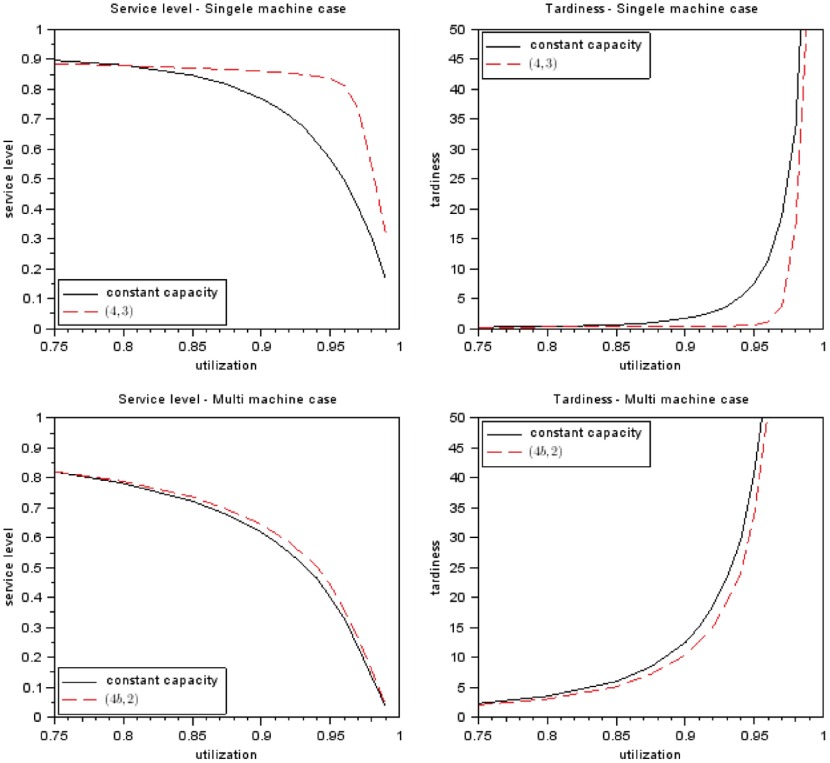
Comparison best capacity setting method with average capacity.Notes: 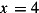: proc time dist, CRL dist; 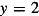: maximum safety; 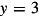: service level target.

Figure 9 shows a comparison of the results gained with constant capacity scenario and method 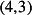 for single-machine production system and 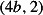 for multi-machine production system. For both situations presented in the following graphs, the basic scenario is compared.

In the single-machine production system, the service level is on average 14% higher and the tardiness on average 60.1% lower when method 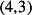 is compared to the constant provided capacity scenario. For the multi-machine production system a service level increase of 2.2% and a tardiness decrease of 13.7% is found in the simulation study. This result shows that flexible capacities, which are provided for medium term capacity setting, can lead to significant improvements in service level and tardiness when the developed capacity setting methods are applied.

For the single and multi-machine production system, the highest improvement potential is found between the planned utilization of 0.85 and 0.97, which is also the range in which a lot of production systems are operating. For low planned utilization values, that the developed methods do not improve the service level and tardiness performance. Hence, in these situations the methods can still be used to decrease the overall provided capacity in the production system. The detailed simulation results (not provided in this paper but available on request) for such low utilization values show that with the best methods – 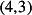 and 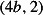 – the provided capacity on average is below one. In this case the developed methods probably lead to lower capacity costs.

Another finding is that the potential to improve service level and tardiness is lower for the multi-machine production system than for the single-machine production system. This effect may result from the higher complexity and higher uncertainty of the multi-machine production system.

### 7.3 Scenario discussion of the best methods

Table 8 gives an overview of the average service level increase and tardiness decrease in the different scenarios for the best identified methods. In the single-machine basic scenario, for example, the average service level increases by 14% when applying method 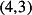 instead of constant provided capacity scenario. The best method for estimating the demanded capacity uses both, information about processing time stochastics and customer demand. This allows the production system to introduce some safety capacity due to process and demand uncertainties. Also, for calculating the provided capacity, the methods providing safety capacity are preferred. Therefore, maximum safety and service level target both perform well.

An interesting finding is that the best methods as found in this study lead in some of the scenarios to a worse overall performance in comparison to constant provided capacity. For the ‘long capacity setting period’ scenario (see Table 5), the reason for the low performance in the multi-machine production system is conjectured to be an increasing demand uncertainty with long periods 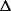, for which capacity has to be set. Taking the above introduced example into consideration, production system with 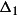 wastes capacity of area one if no additional orders arrive until the next capacity setting. The production system with 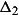, however, wastes capacity of area one and two if no orders arrive until the next capacity setting. Since it is necessary to fulfil Equation (10), too much wasted capacity in some periods leads to a reduced speed of the equipment in later periods.

**Figure 10.**
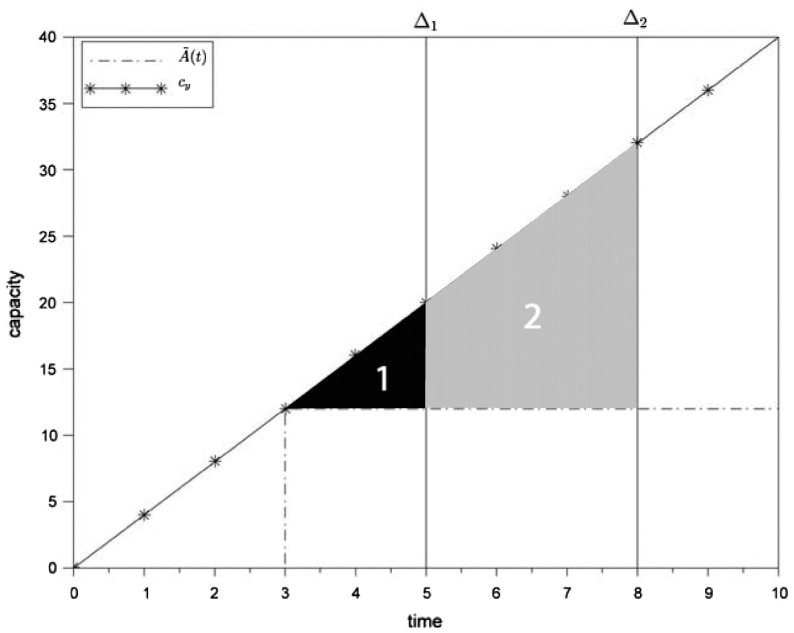
Long capacity setting period and capacity flexibility.

Figure 10 illustrates an example of two production systems at a certain point in time of the timeline (see also Figure 4). The production system is in both cases capable to satisfy 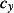. Hence, they differ from capacity setting period 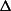, whereby 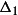 is assuming a short capacity setting period and 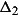 a long capacity setting period.

**Table 8.** Comparison of scenarios.

Scenario	Single-machine case		Multi-machine case	
	*Avg. service level*	*Avg. tardiness*	*Avg. service level*	*Avg. tardiness*
	Increase	Decrease	Increase	Decrease
	[%]	[%]	[%]	[%]
Basic	14.0	60.1	2.2	13.7
High proc-time variation	13.9	56.4	2.5	13.2
Low proc-time variation	12.0	50.9	0.5	4.3
High cust. req. leadtime	8.2	43.4	1.7	12.4
Low customer req. leadtime	7.3	33.6	1.8	10.5
High flexibility	7.8	36.1	0.9	6.3
Low flexibility	5.9	36.9	1.2	8.1
Indefinite flexibility	-7.6	-62.6	-6.9	-57.0
Long capacity setting period	3.4	17.8	-0.5	-2.7
Short capacity setting period	16.3	65.8	2.6	16.7

Notes: 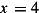: proc time dist, CRL dist; 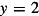: maximum safety; 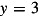: service level target.

A further result based on Table 8 is that for any change in capacity flexibility the performance on both scenario types is worse than in the basic scenario. A too high range of capacity flexibility can lead to overreactions in many situations (see high and indefinite flexibility scenarios). If a backlog exists or the customer requests a high amount of capacity as demonstrated in Figure 10 then the provided capacity it set to the upper bound 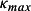. Again, if in some periods the capacity is set to its maximum amount then in later periods it is only possible to use the lower bound 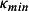 according to Equation 14. However, a too low capacity flexibility gives the developed methods only a narrow range to react on the customer demand. For practical application of these methods, the capacity flexibility used has to be evaluated to determine whether it is still increasing performance or if it is already leading to unnecessary overreactions on single demand peaks.

## 8 Conclusion

In this paper, capacity setting methods are developed to improve service level and tardiness. Information about process uncertainty and/or customer behaviour including the rolling horizon effects of a planning system are implemented in different methods. The results from a simulation study show that the methods 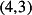 and 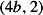 which use information about both the processing time distribution and the customer required lead time distribution lead to the best result. In the single-machine production system an average service level increase (over all experiments for the basic scenario) of 14% and a tardiness reduction of 60.1% have been reached in comparison to a constant provided capacity. For the multi-machine production system the average service level increase is still 2.2% and the average tardiness decrease is 13.7%. Especially in the utilization range of 85% to 97% the developed methods lead to good results. The same methods perform well concerning the two metrics service level and tardiness. The scenarios tested show that too much flexibility in provided capacity leads to overreactions and for this reason to an overall lower performance increase or even to a performance decrease in comparison to the constant provided capacity. Based on the equations developed in this paper the best method can directly be used for practical application to improve logistical performance. Decision makers can decide on which method to apply based on the available data, production system complexity and the available software.

An extensive parameter optimization search for the best method to give some guidelines about optimal parameter sets is postponed to further research.
